# Role of the gut microbiota in complications after ischemic stroke

**DOI:** 10.3389/fcimb.2024.1334581

**Published:** 2024-04-05

**Authors:** Jinwei Zhang, Ling Ling, Lei Xiang, Wenxia Li, Pengnan Bao, Wei Yue

**Affiliations:** ^1^ Clinical College of Neurology, Neurosurgery and Neurorehabilitation, Tianjin Medical University, Tianjin, China; ^2^ Department of Neurology, Tianjin Huanhu Hospital, Tianjin, China

**Keywords:** gut microbiota, ischemic stroke, complication, prognosis, treatment

## Abstract

Ischemic stroke (IS) is a serious central nervous system disease. Post-IS complications, such as post-stroke cognitive impairment (PSCI), post-stroke depression (PSD), hemorrhagic transformation (HT), gastrointestinal dysfunction, cardiovascular events, and post-stroke infection (PSI), result in neurological deficits. The microbiota-gut-brain axis (MGBA) facilitates bidirectional signal transduction and communication between the intestines and the brain. Recent studies have reported alterations in gut microbiota diversity post-IS, suggesting the involvement of gut microbiota in post-IS complications through various mechanisms such as bacterial translocation, immune regulation, and production of gut bacterial metabolites, thereby affecting disease prognosis. In this review, to provide insights into the prevention and treatment of post-IS complications and improvement of the long-term prognosis of IS, we summarize the interaction between the gut microbiota and IS, along with the effects of the gut microbiota on post-IS complications.

## 1 Introduction

Ischemic stroke (IS), a common central nervous system disease, is one of the most severe health problems worldwide, with an annual incidence of 24.9 million patients (Benjamin et al., 2018) and is the second-largest cause of mortality and third-largest cause of disability worldwide (Mendelson and Prabhakaran, 2021). The brain tissue injury in IS is caused by cerebral artery stenosis, occlusion, or acute blood circulation disorders, accounting for 75–85% of all stroke types (Edwards and Bix, 2019; Yang et al., 2022). IS is characterized by suddenness, rapidity, disability, and high mortality (Widimsky et al., 2023). Following the onset of IS, several patients present with various complications, such as post-stroke cognitive impairment (PSCI), post-stroke depression (PSD), hemorrhagic transformation (HT), gastrointestinal dysfunction, cardiovascular events, and post-stroke infection (PSI), which impact disease prognosis, resulting in progressive neurological deficits and high mortality (Lindner et al., 2019; Ułamek-Kozioł et al., 2020; Huang et al., 2022; Zhang et al., 2022; Hede Ebbesen et al., 2023; Wang et al., 2023b).

The gut microbiota refers to all microorganisms, including bacteria, viruses, fungi, and archaea, that colonize the human digestive tract (Benakis et al., 2020). The gut microbiota and its surrounding environment regulate the immune barrier, maintain homeostasis of the gut environment under normal conditions (Victoria et al., 2020), and affect distant organs, including the brain (Delgado Jiménez and Benakis, 2021). The gut microbiota and central nervous system interact through neural, endocrine, and immune mechanisms and form a bidirectional regulatory axis, namely, the microbiota-gut-brain axis (MGBA) (Luan et al., 2019). The gut microbiota also plays a crucial role in the occurrence and development of IS (Wang et al., 2022), which in turn alters the composition of the gut microbiota, which significantly influences the onset of post-IS complications and affects disease prognosis (Pluta et al., 2021).

This review discusses the interactions between the gut microbiota and IS, as well as the impact of the gut microbiota on post-IS complications, to provide insights into the prevention and treatment of post-IS complications and improvement of the long-term prognosis IS.

## 2 Gut microbiota and IS

The human gut microbiota contains trillions of microorganisms, with over 1,000 bacterial species identified and approximately 3 million genes, which is 150 times the size of the human genome (Nam, 2019). The gut microbiota, predominantly composed of Firmicutes and Bacteroidetes (Tyler Patterson and Grandhi, 2020), maintains the integrity of the intestinal epithelial barrier (Ballway and Song, 2021). Pathological alterations in the diversity and abundance of the gut microbiota have been reported to disrupt MGBA signaling, resulting in serious pathophysiological consequences (Chidambaram et al., 2022a). The interaction between the gut microbiota and IS significantly influences the onset, development, and prognosis of IS (Yuan et al., 2021a; Chidambaram et al., 2022b; Han et al., 2023).

The MGBA connects the brain and gut via both direct and indirect pathways, which involve neural pathways, endocrine pathways, the immune system, bacterial metabolites, and host metabolic pathways (Peh et al., 2022). The brain directly communicates with the gut via parasympathetic and sympathetic nerve fibers and indirectly through the stimulation of the enteric nervous system (Browning and Travagli, 2014; Carabotti et al., 2015). In addition, biochemical changes in the brain’s hypothalamic-pituitary-adrenal (HPA) axis can cause changes in intestinal physiology (Wang et al., 2022). The HPA axis activates stress responses that affect intestinal permeability, motility, and mucus production, thereby changing the intestinal environment and affecting the composition and activity of the intestinal microbiota (Wang et al., 2022). After an IS, the proportion of *Firmicutes* and *Bacteroidetes* in the gut microbiota is altered (Hu et al., 2022). The abundance of conditional pathogenic bacteria, such as *Enterobacter* and *Desulfovibrio*, increases (Xia et al., 2019), while that of beneficial short-chain fatty acid (SCFA)-producing bacteria, such as *Blautia*, *Roseburia*, *Bacteroides*, *Lachnospiraceae*, and *Faecalibacterium*, decreases (Li et al., 2019; Tan et al., 2021). Patients with acute IS have reported gut microbiota disorders that last over three weeks, which significantly decreases microbial diversity (Xia et al., 2019); after four weeks, the gut microbiota is gradually restored (Xia et al., 2019). Meanwhile, in patients with mild IS, the abundance of *Enterobacteriaceae* and *Trichospiridae* increases, whereas in patients with severe IS, the abundance of *Ruminococcaceae* and *Christensenaceae* increases (Li et al., 2019).

The immune system, including both adaptive and innate immunity, plays a crucial role in the gut-brain axis, and the gut microbiome plays a key role in brain inflammation, injury, and behavior by regulating the development and function of immune cells in the CNS, regulating peripheral immune responses, and affecting CNS immune activation and the integrity of the blood–brain barrier (BBB) (Milani et al., 2017; Cryan et al., 2019). After focal cerebral ischemia, the levels of pro-inflammatory cytokines interferon-γ (IFN-γ), interleukin-6 (IL-6), and tumor necrosis factor-α (TNF-α) are increased (Muhammad et al., 2023). Following IS, the host immune system is severely suppressed, and the intestinal immune barrier function is disrupted (Brea et al., 2021). This may be related to a decrease in intestinal tight junction protein expression post-IS (Ye et al., 2021), an increase in intestinal epithelial permeability induced by microRNAs (miRNAs) (Wu et al., 2017), and an increase in toxic metabolites that affect the intestinal mucosal epithelium (Kurita et al., 2020; Rustia et al., 2021). After the intestinal barrier function is damaged, pro-inflammatory cytokines are released from the intestine into the circulation, directly communicating with the brain and exacerbating pathological changes (Muhammad et al., 2023). Endotoxins produced by intestinal microorganisms, such as lipopolysaccharides (LPS) and peptidoglycans, enter the bloodstream through the intestinal wall with high permeability and activate the innate immune response of the host, thereby exacerbating inflammatory reactions (Brea et al., 2021). Gut microbiota imbalance in the gut and brain affects the number of lymphocytes, such as γδT cells (Fang et al., 2023), a group of cells with innate immune function mainly located on the surface of the intestinal epithelium (Jiang et al., 2023). Changes in the gut microbiota post-IS trigger pro-inflammatory T-cell responses, increasing the migration of immune cells from the gut to the central nervous system (Jiang et al., 2023). The secretion of IL-17 by T cells leads to the production of chemokines from peripheral medullary cells (monocytes and neutrophils), thereby damaging the blood-brain barrier and inducing neuroinflammation, which exacerbates ischemic brain injury (Samuelson et al., 2019; Wang et al., 2022). In addition, intestinal microorganisms enter the circulation and extraintestinal organs, leading to local and systemic infections (Wang et al., 2023b).

Through this bidirectional communication network, the gastrointestinal tract can also affect brain functions through metabolites produced by the microbiota (Carabotti et al., 2015), which include high levels of acetic, propionic, and butyric acids and low levels of formic, valeric, and caproic acids (Fang et al., 2023). SCFAs promote stroke recovery and exert a protective effect on the intestinal epithelial barrier, thus improving disease prognosis (Hu et al., 2022). Decreased plasma SCFA levels are significantly associated with poor stroke prognosis (Fang et al., 2023). Fecal transplantation of SCFA-producing bacteria or supplementation with SCFAs has been shown to enhance intestinal mucosal integrity and promote the migration of intestinal Tregs to cerebral ischemic areas (Wang et al., 2023c), thereby reducing neuroinflammation and significantly improving neurological deficits (Lee et al., 2020; Sadler et al., 2020). In addition, decreased levels of another gut-derived microbial metabolite, trimethylamine oxide (TMAO), are closely related to the systemic inflammatory response post IS, and early elevated TMAO levels are predictors of poor stroke prognosis (Zhang et al., 2023a).

## 3 Impact of the gut microbiota on Post-IS complications

### 3.1 Gut microbiota and PSCI and PSD

**Figure 1:**
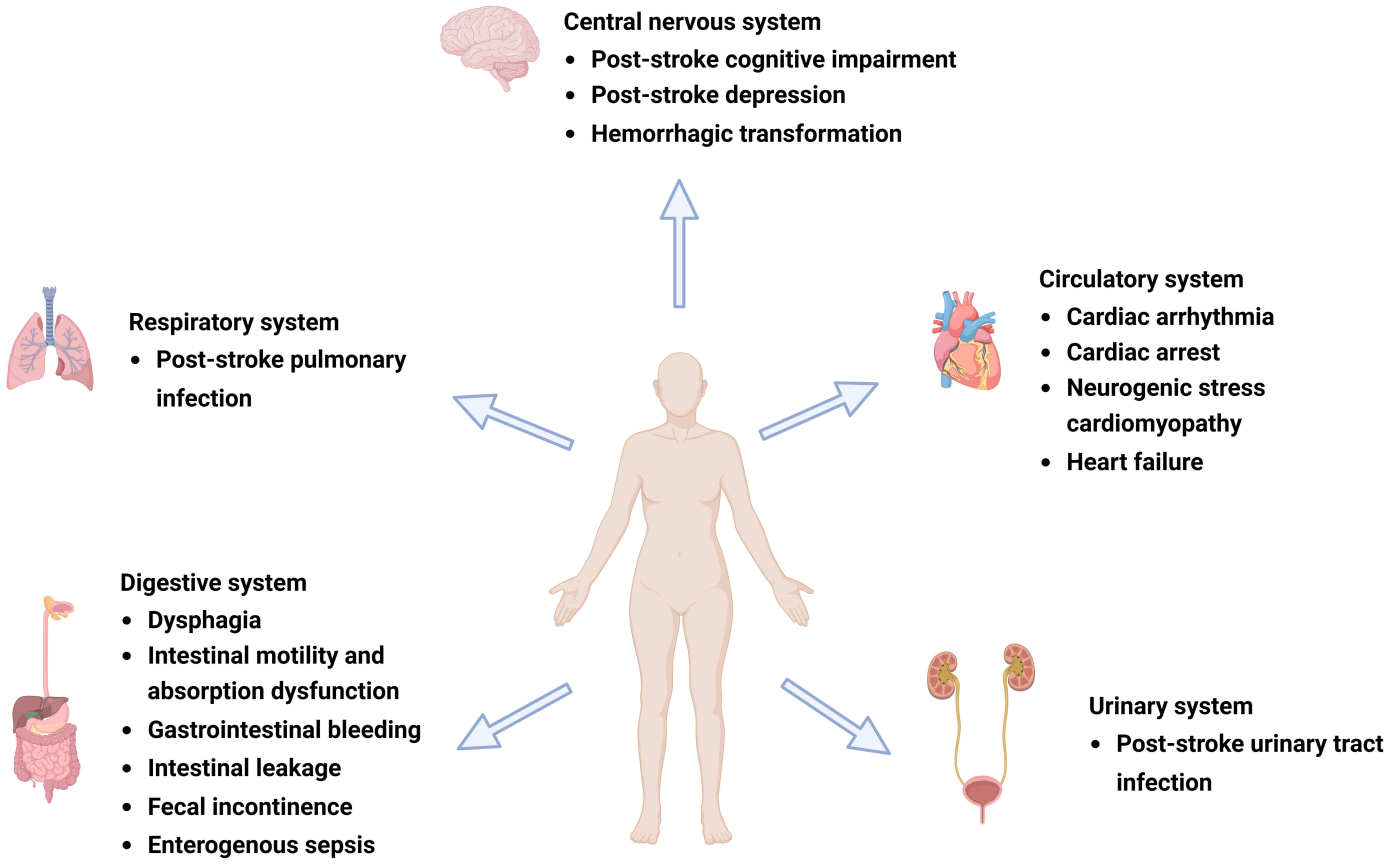
Complications after ischemic stroke (IS). Post-IS complications involve the central nervous, respiratory, circulatory, digestive, and urinary systems and can include post-stroke cognitive impairment (PSCI), post-stroke depression (PSD), hemorrhagic transformation (HT), gastrointestinal dysfunction, cardiovascular events, and post-stroke infection (PSI). Figure generated by BioRender.com.

IS can lead to various neuropsychiatric disorders, such as depression, anxiety, personality changes, mania, and cognitive impairment (Ling et al., 2020), of which PSCI and PSD are among the most common (Caso et al., 2009; Goyal et al., 2020) and are indicative of poor prognosis and high mortality (Jiang et al., 2021). Cognitive dysfunction is closely associated with depression; these diseases often interact with each other (Douven et al., 2018) and coexist in patients with stroke (Douven et al., 2018). A growing body of evidence suggests that gut microbiota dysbiosis affects the physiological, behavioral, and cognitive functions of the brain through various neural, immune, endocrine, and metabolic pathways via MGBA, which plays an important role in PSCI and PSD (Li et al., 2018; Ling et al., 2020; Kang et al., 2021) (**Figure 1**).

The diversity of the gut microbiota is reduced in patients with PSCI and PSD compared with that in healthy individuals, suggesting that higher gut microbiota diversity may reflect a relatively healthy state following acute IS (Smith and Wissel, 2019; Jiang et al., 2021). In addition, the gut microbiota composition in patients with PSCI is significantly different, as manifested by elevated *Fusobacterium* species (Yu et al., 2017), a gram-negative non-spore pathogen that induces an imbalance in the intestinal microenvironment, creates a pro-inflammatory environment in the body, and causes immune evasion of certain pathogenic bacteria (Yu et al., 2017). *Fusobacterium* has been reported to exacerbate cognitive impairment by promoting inflammation in mice (Yu et al., 2017). Gut microbiota in patients with post-stroke cognitive impairment and depression (PSCCID) is characterized by elevated levels of *Proteobacteria*, including γ-*Protobacteria*, *Enterobacteriales*, and *Enterobacteriaceae* (Ling et al., 2020). The number of γ-Proteobacteria and *Enterobacteriaceae* is negatively correlated with the Montreal Cognitive Assessment Scale score, suggesting that these gut bacteria are associated with cognitive impairment (Ling et al., 2020). The number of *Bifidobacteria* in the feces of patients with PSD decreases, whereas the numbers of *Enterococcus faecalis* and *Escherichia coli* increase (Kang et al., 2021). *E. faecalis* and *E. coli* are important opportunistic pathogens of the gastrointestinal tract (Sapountzis et al., 2020). *Bifidobacteria* are beneficial bacteria in the human gut that inhibit the proliferation of pathogenic bacteria, exert potent antidepressant effects, and regulate the microbial community (Tian et al., 2020).

Dysregulated gut microbiota exacerbate the damage to gut barrier function, leading to an excessive release of local inflammatory factors in the gastrointestinal tract (Pu et al., 2023), which is closely associated with the occurrence and exacerbation of depressive symptoms (Buglione-Corbett et al., 2018). Higher serum levels of IL-1, IL-2, IL-6, and high-sensitivity C-reactive protein (hsCRP) have been reported in patients with PSD than in those without PSD, suggesting the involvement of inflammatory cytokines in the pathological mechanisms of PSD and inflammatory responses (Kang et al., 2021). In patients with PSD, the levels of *E. faecalis* and *E. coli* are positively correlated with IL-1, IL-2, IL-6, and hsCRP levels, whereas those of *Bifidobacterium* are negatively correlated with inflammatory factors (Kang et al., 2021). Disruption of the gut microbiota in patients post-IS promotes the occurrence and development of PSD by increasing intestinal permeability, promoting intestinal bacterial translocation, activating downstream immune responses, and exacerbating inflammatory reactions (Kang et al., 2021). In addition, gut microbiota, especially those containing LPS, including Proteobacteria and *Enterobacteriaceae*, are involved in chronic mild inflammatory reactions (Hiippala et al., 2018). Neuroinflammation is associated with cognitive impairment (Passamonti et al., 2019). Therefore, inflammation-related gut bacteria is potentially involved in the development of PSCCID.

Previous studies have demonstrated a significant correlation between SCFA deficiency and PSCI (Dalile et al., 2019). SCFAs interact with free fatty acid receptors, inhibit histone deacetylases, enter the brain through the blood-brain barrier, affect microglia, reduce nervous system inflammation, and play key roles in MGBA (Dalile et al., 2019; Parada Venegas et al., 2019). The number of bacteria producing SCFAs in patients with PSCCID, such as Trichospiridae, is lower than that in patients with non-PSCCID (Ling et al., 2020).

In addition, probiotic interventions composed of lactobacilli and Bifidobacteria improved post-stroke emotional state, including anxiety and depression, but had no significant effects on cognitive function (Liu et al., 2020). Postbiotics, also referred to as metabiotics, biogenics, or cell-free supernatants (CFSs), are bacterial fermentation metabolites and soluble factors secreted by live bacteria or released after bacterial lysis, such as SCFAs, enzymes, teichoic acids, endo- and exopolysaccharides, cell surface proteins, vitamins, plasmalogens, and organic acids (Vallianou et al., 2020; Sorboni et al., 2022). The most important SCFAs are acetate, propionate, and butyrate (De Vadder et al., 2014). By supplementing high doses of SCFAs or probiotics that produce SCFAs instead of traditional doses of *lactobacilli* and *Bifidobacteria*, PSCI may be effectively improved (Liu et al., 2020). Animal experiments have shown that SCFAs may improve cognitive function by inhibiting amyloid-β protein aggregation *in vitro* (Ho et al., 2018). Additionally, fecal bacterial transplantation or supplementation with new probiotics such as *Akkermansia* may improve PSCI (Liu et al., 2020). Exercise training following stroke increases the expression of *lactobacilli* and *Bifidobacteria*, improves the function of the intestinal microbiota, reduces the expression of inflammatory factors such as IL-2 and IL-6 in intestinal and brain tissues, promotes the transformation of microglia from the M1 to M2 type, inhibits neuroinflammatory responses, and improves depression (Tian et al., 2019; Yang et al., 2021).

### 3.2 Gut microbiota and HT

HT, a common complication of acute IS, leads to the progression of the condition and exacerbation of neurological damage and increases the risk of other complications (Hafez et al., 2015). The pathological mechanism underlying HT has been linked to the disruption of the blood-brain barrier, oxidative stress, inflammatory response, and intestinal microbiota disorders (Huang et al., 2022).

Post-stroke HT significantly increases the number of anaerobic bacteria such as *Actinobacteria*, *Proteobacteria*, and *Verrucomycetes*, and *Proteobacteria* enrichment has been observed in the feces of HT rats (Huang et al., 2022). Specific changes in the gut microbiota may be influenced by the levels of certain organic acids and matrix metalloproteinase-9 (MMP-9) (Huang et al., 2022). The early BBB disruption modulated by increased expression of MMPs is closely associated with HT events in ischemic stroke (Liu et al., 2009). Research has found that the levels of MMP-9 in HT rats significantly increased, and further analysis revealed a negative relationship between MMP- 9 and total SCFA and propanoic acid concentrations (Huang et al., 2022). In addition, the relative abundance of *Holdemania* and *Collinsella* was negatively correlated with MMP- 9 concentration, while *Allobaculum*, *Erysipelotrichi*, *Erysipelotrichales*, and *Erysipelotrichaceae* were positively correlated with MMP- 9 levels (Huang et al., 2022). Oxidative stress plays an important role in the pathological process of HT pathogenesis. Some scholars have proposed that hyperglycemia increases the formation of free radicals and reduces oxygen production in transient ischemic rats (Li et al., 1999). On the other hand, oxidative stress also reduced the oxygen content in the intestinal tract and caused perturbed disruption of cecal microbiota in mice (Zhao et al., 2021). Thus, HT induced by hyperglycemia may inhibit the oxygen formation in the intestinal tract and increase the production of anaerobic bacteria (Huang et al., 2022).

The levels of SCFAs, especially butyric and valeric acids, are lower in the guts of HT than in those of non-HT rats, as is the number of microorganisms producing SCFAs (Huang et al., 2022). Butyric acid maintains intestinal epithelial integrity, regulates the immune system, and alleviates inflammatory response (Sadler et al., 2020). Furthermore, SCFA levels following HT are significantly correlated with inflammatory cytokines, including TNF- α, IL-1 β, and IL-17, which have a significant impact on the prognosis of IS (Huang et al., 2022).

### 3.3 Gut microbiota and post-IS gastrointestinal dysfunction

Over 50% of patients with IS experience gastrointestinal complications, including swallowing difficulties, intestinal motility and absorption disorders, gastrointestinal bleeding, intestinal leakage, fecal incontinence, and intestinal septicemia (Durgan et al., 2019; Long et al., 2022). Extensive research in genomics, metabolomics, and proteomics has revealed that the gut microbiota is involved in several pathophysiological events following stroke, and patients with IS with gastrointestinal complications often have a poor prognosis, high mortality rate, and neurological function deterioration (Tuz et al., 2022; Zhao et al., 2023).

**Figure 2:**
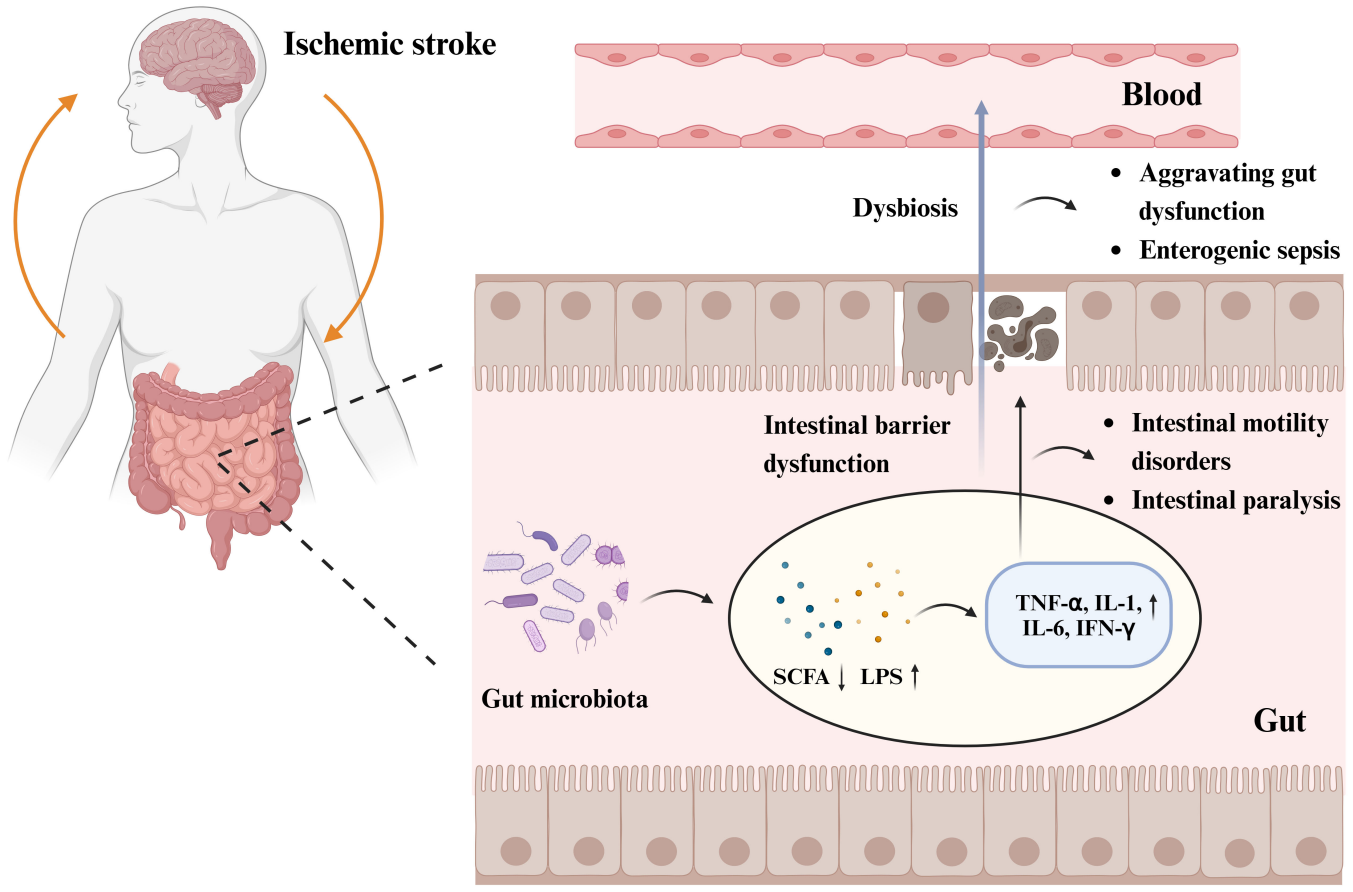
Gut microbiota and post-stroke gastrointestinal dysfunction pathogenesis. Increased abundance of gut microbiota, decreased beneficial metabolites short-chain fatty acids (SCFAs), and increased LPS of Gram-negative bacteria post IS, triggered an inflammatory response with increased expression levels of inflammatory factors such as tumor necrosis factor-α (TNF-α), interleukin (IL)-1, IL-6, and cytokine interferon-γ (IFN-γ). The cytotoxic effects of inflammatory substances lead to intestinal microvillus damage and reduced expression of intestinal epithelial tight junction proteins, triggering intestinal epithelial barrier dysfunction and intestinal leakage, which can lead to intestinal motility disorders, intestinal paralysis, and other gastrointestinal complications. Impairment of intestinal barrier function allows inflammatory cytokines, bacteria, and toxic intestinal metabolites to cross the damaged intestinal epithelial barrier and enter the circulatory system, exacerbating gastrointestinal dysfunction and even triggering enterogenic sepsis. Figure generated by BioRender.com.

Following stroke, the gut microbiota is dysregulated, and the abundance of gram-negative *Enterobacteriaceae* increases (Huang and Xia, 2021). Multiple studies have shown a correlation between intestinal epithelial barrier dysfunction, intestinal leakage in patients with IS, and changes in the gut microbiota (Zhang et al., 2022), which can lead to intestinal villous epithelial damage, reduced mucus and expression of intestinal tight junction proteins, increased intestinal permeability, and intestinal sepsis (Zhao et al., 2023). The disruption of intestinal epithelial barrier function is associated with the LPS of gram-negative bacteria, metabolites of dominant bacterial populations such as SCFAs and TMAO, and intestinal inflammatory factors, including TNF-α, IL-1, IL-6, and nitric oxide synthase (Cheng et al., 2018; Chen et al., 2019c; Yu et al., 2021). Opportunistic pathogens produce harmful substances, such as LPS (Kurita et al., 2020), an endotoxin cell wall component of gram-negative bacteria that triggers inflammatory reactions by mediating the Toll-like receptor (TLR) 4/MyD88 signaling pathway (Singh et al., 2016). The cytotoxic effects of inflammatory substances damage the intestinal microvilli, which affects the expression levels of intestinal tight junction proteins, exacerbates damage to the intestinal barrier function (Kurita et al., 2020), and leads to intestinal leakage, thereby allowing inflammatory cytokines, bacteria, and toxic intestinal metabolites to penetrate the damaged intestinal epithelial barrier and enter circulation (Ye et al., 2021). The levels of LPS, D-lactate, zonulin, TNF-α, IFN-γ, and IL-6 are increased in the serum of macaques with IS compared with those in healthy macaques (Chen et al., 2019c). Endotoxemia and bacterial translocation can exacerbate gastrointestinal complications, such as intestinal bleeding, motility disorders, and intestinal paralysis (Wang et al., 2023a). The metabolic product SCFAs of the gut microbiota are crucial to maintaining intestinal bacterial balance, epithelial functional integrity, immunology, and inflammation. Reduction in SCFAs post IS has also been confirmed (Zou et al., 2022); SCFAs promote the development of the intestinal barrier and protect it against LPS damage by inhibiting NOD-like receptor thermal protein domain-associated protein 3 (NLRP3) inflammasomes (**Figure 2**).

The intestinal immune system comprises multiple immune tissues and cells that cooperate under physiological conditions to resist pathogen invasion and maintain immune homeostasis (Pu et al., 2023; Zhao et al., 2023). Under normal physiological conditions, B cells differentiate into IgA-producing B cells in the presence of T cells to eliminate toxins and pathogens (Suzuki and Nakajima, 2014). In a rat stroke model, pre-ischemic stress significantly reduces large intestinal IgA levels and bacterial translocation (Caso et al., 2009). In addition, a decrease in the number of B cells in the small intestine of mice post-IS has been reported (Oyama et al., 2018); therefore, IS may adversely affect the homeostasis of the local and systemic immune systems in the intestine, impairing local antibacterial defense and gastrointestinal complications (Oyama et al., 2018).

The single or combined use of dietary interventions, probiotics, microbial metabolites, fecal microbiota transplantation, and traditional Chinese medicine can prevent and improve gastrointestinal complications following stroke (Liu et al., 2020; Wei et al., 2023) (**Table 1**). In patients with stroke, early enteral nutrition combined with probiotic therapy, such as *Lactobacillus* and *Bifidobacterium*, can improve nutritional status, replenish intestinal microbiota, stabilize intestinal barrier function, improve immune tolerance, and reduce the incidence of nutritional diarrhea (Mao et al., 2022). Probiotics inhibit the adhesion of opportunistic pathogens to the intestinal wall, prevent excessive growth of pathogens and the invasion of foreign pathogens, reduce apoptosis of intestinal epithelial cells caused by pathogens, protect the intestinal mucosal barrier, and inhibit bacterial migration (Martínez-Guardado et al., 2022). Probiotics can also produce bioactive compounds, including bacteriocins, organic acids, vitamins, and neurotransmitters, reduce oxidative stress and inflammatory cytokines, and improve intestinal and systemic immune functions (Chen et al., 2022).

**Table 1:** Treatment strategies of gut microbiota in gastrointestinal complications after stroke.

Strategies		Mechanism	Organism	Reference
Dietary interventions	High-fiber diets	1. Increase SCFA levels in the blood and reduce intestinal inflammatory response. 2. Alleviate intestinal barrier dysfunction.	Humans	(Wurtman, 2008)
Probiotics	*Lactobacillus* *Bifidobacterium*	1. Inhibits the adhesion of opportunistic pathogens to the intestinal wall and prevents the excessive growth and invasion of foreign pathogens. 2. Maintains intestinal microbiota balance, reduces pathogen-induced intestinal epithelial cell apoptosis, protects intestinal mucosal barrier, and inhibits bacterial translocation. 3. Produces bioactive compounds, including bacteriocins, organic acids, vitamins, and neurotransmitters. 4. Reduces oxidative stress and inflammatory cytokine levels, produces anti-inflammatory compounds, and improves intestinal and systemic immune function.	Humans	(Mao et al., 2022)
Prebiotics	Lactulose	1. Improves intestinal microbiota imbalance and repairs damage in the intestinal barrier. 2. Increases anti-inflammatory factors in the brain and intestines and inhibits inflammatory responses.	C57 mice	(Yuan et al., 2021b)
	BARLEYmax	1. Enhances the abundance of butyric acid-producing bacteria and increases butyric acid in the gastrointestinal tract. 2. Moderately increases the abundance of Bacteroidetes and reduces the abundance of *Clostridium*.	Humans	(Akagawa et al., 2021)
Postbiotics	IPA	1. Increases the number of probiotics and reduces the abundance of harmful bacteria. 2. Protects the intestinal barrier integrity.	C57 mice	(Xie et al., 2022)
Fecal bacteria transplantation	Fecal bacteria rich in SCFAs	1. Remodel the intestinal microenvironment. 2. Enrich beneficial lactobacilli. 3. Repair intestinal leakage.	SD rats	(Chen et al., 2019b)
Traditional Chinese Medicine	TQHXD	1. Regulates gut microbiota, reduces excessive increase of *Bacteroides*, and controls abnormal changes in certain microbiota. 2. Improves inflammatory response caused by T-cell imbalance. 3. Restores the function of the intestinal barrier.	SD rats	(Spence, 2018)
	PLR+ CXR	1. Remodels intestinal microenvironment. 2. Increases claudin-5 and ZO-1 to protect the intestinal barrier. 3. Reduces intestinal microbiota translocation.	SD rats	(Chen et al., 2019a)
Other	EA+ iPSC EVs	1. Increase the number of neurons significantly. 2. Downregulate the expression of IL-17 in brain and colon tissues and upregulate IL-10 levels. 3. Regulate the composition and diversity of gut microbiota.	C57 mice	(Zhang and Yao, 2023)

SCFAs, short-chain fatty acids; IPA, indole-3-propionic acid; C57, C57BL/6; SD, Sprague-Dawley; TQHXD, Tong-Qiao-Huo-Xue Decoction; PLR, Pueraria lobata root; CXR, Chuanxiong Rhizoma; EA, electroacupuncture; iPSC EVs, induced pluripotent stem cell-derived extracellular vesicles; IL, interleukin.

Prebiotics, especially carbohydrates, affect the production of SCFAs and mucin and regulate local inflammatory responses in gut-associated lymphoid tissue (GALT) (Markowiak and Śliżewska, 2017). Lactulose Supplementation (a common prebiotic) can repair intestinal barrier damage, alleviate intestinal microbiota imbalance, and improve neurological function after stroke (Yuan et al., 2021b). Furthermore, the fiber-rich barley, BARLEYmax, a prebiotic, has been reported to increase the abundance of butyric acid-producing bacteria and butyric acid levels in the gastrointestinal tract (Akagawa et al., 2021). Combining probiotics and prebiotics has a synergistic effect (Chen et al., 2019a); indeed, the combination of inulin and SCFA-producing bacteria increased SCFA production in mice following stroke and improved neurological deficit scores and behavioral outcomes compared with pre-administration of SCFA-producing bacteria (Chen et al., 2019a). Therefore, probiotics and prebiotics improve the microbiota composition and gastrointestinal function, thereby positively affecting patient prognosis (Wei et al., 2023).

Postbiotics also confer physiological benefits to the host. The oral administration of indole-3-propionic acid to MCAO mice has been shown to increase the number of probiotics, reduce harmful bacteria, and protect intestinal barrier integrity (Xie et al., 2022; Zhou et al., 2023). In addition, including fiber, butyrate, or butyrate-producing probiotics in the diet can reduce intestinal inflammation; affect tight junction proteins (Wurtman, 2008; Liu et al., 2020); upregulate the expression levels of ZO-1, occlusion, and claudin4 to maintain intestinal mucosal integrity; affect epithelial oxygen consumption; maintain the stability of hypoxia-inducible factors; improve intestinal barrier dysfunction; reduce adverse prognosis related to IS (Wang et al., 2021). Transplantation of SCFA-rich fecal microbiota can reshape the gut microbiota, enrich beneficial lactobacilli, and repair intestinal leakage (Chen et al., 2019b).

The traditional Chinese medicine Tong-Qiao-Huo-Xue Decoction can control changes in the bacterial community post IS, reduce excessive increases in *Bacteroides*, control abnormal changes in the abundance of specific bacterial communities, improve the inflammatory response caused by T-cell imbalance, and restore the function of the intestinal barrier (Spence, 2018). Combining Pueraria lobata root and Chuanxiong Rhizoma (CXR) in treating IS in rats has been shown to alleviate intestinal microbiota imbalance and damage to the brain-intestinal barrier, effectively improving neurological function (Chen et al., 2019a). Electroacupuncture induced pluripotent stem cell-derived extracellular vesicles (iPSC EVs), while electroacupuncture combined with iPSC EVs increased the number of neurons, inhibited inflammation in MCAO mice, alleviated colon injury, regulated the gastrointestinal microbiota, and reduced the occurrence of gastrointestinal complications post-IS (Zhang et al., 2023b).

### 3.4 Gut microbiota and post-IS cardiovascular events

Cardiovascular complications are the second leading cause of death after stroke (Yoshimura et al., 2008). Despite the best treatment based on guidelines for patients with transient ischemic attacks and stroke (Haghikia et al., 2018), in the initial 3 months post-acute IS, 19.0% of patients experience at least one serious cardiac adverse event; 28.5% of patients have a left ventricular ejection fraction below 50%; 13–29% suffer from cardiac systolic dysfunction (Chen et al., 2017b). Cardiac complications of stroke that lead to mild recoverable injury, lifelong heart problems, or even death include congestive heart failure, neurogenic stress cardiomyopathy, Takotsubo cardiomyopathy, cardiac arrest, and arrhythmias (Samuels, 2007; Holmqvist et al., 2015). Following IS, an increase in intestinal permeability promotes an inflammatory response, and systemic inflammation increases intestinal permeability (Pu et al., 2023). The translocation of bacteria and endotoxins into the bloodstream increases the levels of pro-inflammatory cytokines, while systemic inflammation induces or exacerbates cardiac dysfunction (Masenga et al., 2023).

Analysis of cardiovascular event data in patients with stroke revealed a dose-dependent correlation between elevated levels of intestinal microbiota metabolites (TMAO) and an increased risk of cardiovascular events following the initial stroke episode (Haghikia et al., 2018). Receiver operating characteristic curve analysis further showed that TMAO is a predictor of the 1-year risk of cardiovascular death in patients with IS (Haghikia et al., 2018). Foods rich in specific nutrients with trimethylamine groups, such as phosphatidylcholine, choline, and carnitine, are degraded by trimethylamine lyases encoded by the gut microbiota in the intestine, and, upon absorption, are further metabolized into TMAO by liver flavin-containing monooxygenases (FMO), especially FMO3 (Kasahara and Rey, 2019). TMAO is associated with the development of cardiac metabolic diseases and atherosclerosis (Zhang and Yao, 2023). The atherogenic effects of microbial-dependent TMAO include enhanced cholesterol accumulation of macrophages and formation of foam cells, pro-inflammatory changes in the arterial wall, platelet hyperreactivity, and enhancement of the potential for arterial thrombosis (Hemmati et al., 2023; Zhang and Yao, 2023).

In mice, TMAO enhanced the development of atherosclerosis by increasing the expression of the scavenger receptor CD36 and scavenger A1 receptor SR-A1 on macrophages, inhibiting the reverse transport of cholesterol and forming foam cells in atherosclerotic lesions, which increased lipid accumulation in vascular wall macrophages (Kasahara and Rey, 2019; Canyelles et al., 2023). This was inhibited following treatment with small-molecule inhibitors produced by microbial trimethylamine (Wang et al., 2015). In animal models, FMO3 inhibition reduced both TMAO and atherosclerosis (Shih et al., 2015).

In addition, TMA activates mitogen-activated protein kinases, extracellular signal-related kinases, and nuclear factors in the endothelial cells-κB signal cascade to promote vascular inflammation (Hemmati et al., 2023). Direct injection of TMAO into rodent models has been shown to promote the activation of aortic endothelial cells and upregulation of adhesion proteins (Hemmati et al., 2023). Studies on cultured endothelial cells have shown that TMAO can activate the NLRP3 inflammasome, which adversely affects atherosclerosis (Chen et al., 2017a). LPS receptors CD14 and Fc γ III receptor CD16^+^ monocytes are a subgroup with specific pro-inflammatory functions; blood TMAO concentration is positively related to the level of pro-inflammatory intermediate CD14^++^CD16^+^ monocytes (Yang et al., 2023). Intermediate CD14^++^CD16^+^ monocytes secrete a large amount of inflammatory cytokines, such as TNF-α, to promote inflammatory responses (Oktaviono et al., 2023). The high expression of adhesion molecules, such as CD162/P-selectin glycoprotein ligand-1 (PSGL-1) and myeloperoxidase, the primary source of reactive oxidants in the innate immune response, in intermediate monocytes contributes to their thrombogenic and atherogenic properties (Wildgruber et al., 2016). In patients with IS, an increase in the number of intermediate monocyte subsets is closely associated with the occurrence of cardiovascular events following cerebral infarction. In addition to promoting an increase in pro-inflammatory monocyte levels, gut microbiota can directly lead to platelet hyperreactivity and increase the release of intracellular stored Ca2+ through the generation of TMAO, thereby increasing the risk of thrombosis (Nesci et al., 2023), and the incidence of cardiovascular disease post-IS.

### 3.5 Gut microbiota and PSI

PSI is reported in up to half of patients with stroke; pneumonia and urinary tract infections are the most common PSIs (Westendorp et al., 2011; Shim and Wong, 2018). PSI is also the leading cause of readmission and death in patients and associated with a poor prognosis (Kaur et al., 2019). The occurrence of pulmonary or urinary tract infections post-stroke is attributed to difficulty in swallowing, advanced age, long-term immobility, or the result of surgeries such as insertion of nasogastric tubes, venous catheters, catheters, or mechanical ventilation. Opportunistic pathogens in hospitals are also sources of infection (Mao et al., 2019). However, the integrity of the intestinal barrier and translocation of intestinal bacteria post-IS may be linked to PSI pathogenesis (Zbesko et al., 2021).

Translocation of gut microbiota and immune dysfunction play crucial roles in the occurrence and development of PSI (Stanley et al., 2018; Ghelani et al., 2021). Stroke induces immune suppression, disrupting cytokine homeostasis, weakening peripheral immune defense, reducing host defense against bacteria, increasing host susceptibility, promoting bacterial migration to the lungs, and exacerbating lung tissue damage (Zhang et al., 2021). Additionally, a decrease in white blood cells and lymphocytes has been observed in the peripheral blood following stroke, with the most significant decrease occurring on the third day (Li et al., 2020). Stroke may also activate the hypothalamic-pituitary-adrenal system through glucocorticoids, inducing peripheral spleen atrophy and leading to impaired lymphocyte production and a lack of natural killer cells, thereby suppressing peripheral immunity (Zhang et al., 2021).

Changes in the gut microbiota composition increase susceptibility to respiratory diseases, promote bacterial migration, and induce PSI (Wattoo et al., 2021). The bacteria detected in the blood, sputum, and urine of patients with IS are common microorganisms residing in the human gut, including *E. coli*, *Enterococcus*, and *Morganella morganii* (Kishore et al., 2018, Kishore et al., 2019). Following stroke, the number of bacteria in the lungs significantly increases, however decreases in the ileum and colon (Hede Ebbesen et al., 2023). The diversity of the microbiota significantly changes, while the composition of the lung tissue and gut microbiota remains similar, indicating that the bacteria disseminated in the lungs are likely to originate from the gut (Hede Ebbesen et al., 2023). The ileum is most sensitive to intestinal permeability post-stroke, subsequently peaking after 3 h, facilitating the entry of intestinal bacteria into surrounding tissues and organs from the intestine and increasing the risk of post-stroke pneumonia (Shim and Wong, 2018).

Additionally, the sympathetic nervous system is involved in the alternation of intestinal permeability post-stroke (Doyle and Buckwalter, 2017). The activation of β-adrenergic receptors post-stroke triggers the destruction of the intestinal barrier and immune suppression, leading to the spread of bacteria in the intestine and the occurrence of infections (Doyle and Buckwalter, 2017). Data obtained from PSI mice indicated that microorganisms residing in the intestine could be detected in the blood and lymph nodes; bacteria from the intestine have also been observed in the liver and spleen (Hede Ebbesen et al., 2023). Therefore, various pathways are potentially involved in the direction of bacterial migration to the lungs post-stroke, including direct transmission from the small intestine to the lungs through the blood and lymphatic systems or indirect migration to lung tissue through the liver (Hede Ebbesen et al., 2023).

SCFAs play an important role in systemic circulation and immune regulation in the brain, possibly affecting PSI (Sadler et al., 2020; Haak et al., 2021), while lower abundance of butyrate-producing bacteria within 24h of hospital admission was an independent predictor of enhanced risk of post-stroke infection (Lee et al., 2019). Butyrate can enhance the antibacterial activity of monocytes and macrophages and the body’s antibacterial activity against respiratory pathogens (Haak et al., 2018) and exert immune regulation by inhibiting histone deacetylase and the mammalian target of rapamycin signals in circulating white blood cells, enabling the host to resist invading pathogens and produce protective effects (Schulthess et al., 2019). Butyrate-producing bacteria can produce other metabolites, such as SCFAs, indoles, and desaminotyrosine, which help to resist infections (Haak et al., 2021), upregulate Tregs, help to suppress the post-ischemic inflammatory response of residents and invading inflammatory cells, and improve PSI (Sadler et al., 2020).

Following IS, the use of antibiotics is a promising strategy to reduce PSI; however, large-scale clinical studies have yet to confirm this (Westendorp et al., 2018). Antibiotics can disrupt beneficial gut microbiota, potentially exacerbating changes therein caused by IS and amplifying inflammatory responses (Singh et al., 2016; Ghelani et al., 2021). In addition, blocking the activation of the sympathetic nervous system with propranolol can prevent increased intestinal permeability, which suggests β-adrenergic receptor antagonists as a potential therapeutic approach (Westendorp et al., 2016).

## 4 Conclusion

IS remains one of the most challenging diseases. Gut microbiota plays a vital role in post-IS complications such as PSCI, PSD, HT, gastrointestinal dysfunction, cardiovascular events, and PSI via several mechanisms, such as microbial diversity changes, immune regulation, and endocrine regulation, utilizing MGBA bidirectional signal transduction. However, the pathological mechanisms by which various microbiotas affect the prognosis of IS require further investigation. Several treatment methods aimed at improving the physiological function and disease progression of patients with IS and the gut microbiota, such as microbiota transplantation, supplementation with probiotics, and gut microbiota metabolites, have shown beneficial effects. Further research on the mechanism of action of gut microbiota and targeted treatment methods may provide novel insights into preventing and improving post-IS complications.

## Author contributions

JZ: Methodology, Software, Supervision, Validation, Writing – original draft, Writing – review & editing. LL: Validation, Writing – review & editing. LX: Supervision, Writing – review & editing. WL: Writing – review & editing. PB: Writing – review & editing. WY: Funding acquisition, Supervision, Validation, Visualization, Writing – review & editing.
